# Regional with urban–rural variation in low birth weight and its determinants of Indian children: findings from National Family Health Survey 5 data

**DOI:** 10.1186/s12884-023-05934-6

**Published:** 2023-08-28

**Authors:** Ramendra Nath Kundu, Anushka Ghosh, Birshikha Chhetri, Indranil Saha, Md. Golam Hossain, Premananda Bharati

**Affiliations:** 1 Former Research Fellow, Department of Anthropology, West Bengal State University, Kolkata, West Bengal 700126 India; 2 Junior Research Fellow, Indian Council of Medical Research-Centre for Ageing & Mental Health, Kolkata, West Bengal India; 3 Scientist E, Indian Council of Medical Research-Centre for Ageing & Mental Health, Kolkata, West Bengal India; 4 Health Research Group, Department of Statistics, University of Rajshahi, Rajshahi, 6205 Bangladesh; 5 Former Professor and Head, Biological Anthropology Unit, Indian Statistical Institute, Kolkata, West Bengal India

**Keywords:** Birth weight, Under-five children, Wealth index, Public health, SDG

## Abstract

**Background:**

Low birth weight is a key indicator for child health, especially a concern in low-middle-income countries. However, health and medically-related reforms are being actively implemented in some middle-income countries like India. Identifying low birth weight (LBW) babies with their determinants across the whole country is essential to formulate regional and area-specific interventions. The objective of this study was to find out the burden and determinants of LBW on the regional and residential (rural–urban) divisions of India.

**Methods:**

The present study was based on the NFHS-5 dataset (2019–21), a nationally representative survey in India. A total of 209,223 births were included in this study. A newborn weighing less than 2500 g was considered as LBW. According to the objectives, we used frequency distribution, chi-square test and binary logistic regression analysis for analysing the data.

**Results:**

About 18.24% of the babies were LBW in India, significantly higher in rural areas than in urban areas (18.58% vs 17.36%). Regionally prevalence was more frequent in western (20.63%) and central (20.16%) rural areas. Regarding maternal concerns, in the eastern and southern regions of India, mothers aged 25–34 were less likely to have LBW children than mothers aged 35–49 years. It was found that the risk of LBW was more likely among the children born out of unintended pregnancies in almost all regions except for eastern part. In rural India, women who delivered children at home were more likely to have LBW children in India (AOR = 1.19, CI: 1.12–1.28, *p* < 0.001) and its central, northern, and southern regions than those who gave birth in institutions. The study indicates that LBW coexists with lower maternal education levels and poor household wealth index across all regions. About 58% and 57% of cumulative effects of independent variables on LBW can be distinguished in urban and rural India, respectively.

**Conclusions:**

Targeted-specific strategies need to be undertaken as per region and geographical variations. Then only India should be able to decline LBW as proposed by National Health Policy.

## Introduction

The birth of a healthy baby signifies an essential benchmark for the survival, health, and nutrition of new-born life. Lower the birth weight, the higher the risk of survival along with the risks associated with the growth and development of a child [1, 2]. The risks and consequences of low birth weight are short-term and long- term continues from childhood to adulthood, leading to stunted growth, impaired mental development, and increased risk of chronic diseases later in life [3–5]. Furthermore, it also implies the health status of a mother before and after childbirth [6]. In the contemporary context of public health and community development, low birth weight has emerged as a key indicator determining child and maternal health [2].

A newborn weighing less than 2500 g is considered low birth weight (LBW) [7]. In 2015, the UNICEF-WHO estimated 20.5 million LBW globally, which accounted for 15 to 20% of all newborns worldwide. Out of which, developing countries contributed 95.6% in the global context [7, 8]. It is a serious concern in low and middle-income countries (LMICs) [9]. Region-wise, LBW is reported to be highest (28%) in South Asia compared to 13% and 9% in sub-Saharan Africa and Latin America, respectively [8]. India alone covers nearly 40% of the LBW babies in developing countries, and this data has been criticised as partial and unreliable data [10].

The underreporting of births at home and small health clinics may have resulted in the underrating LBW [11]. In 2012, the 65th World Health Assembly passed a resolution setting a target of reducing low birth weight by 30% [7]. LBW is also associated with an increase in infant mortality in India [4]. According to the National Family Health Survey (NFHS-4), 18% of Indian children under five were born with LBW [12]. Mothers in LMICs often lack access to sufficient nutrition and health care [13]. This contributes to a greater risk of LBW in India [14]. The Sustainable Development Goals (SDGs) to lower infant mortality by 2030 have been reaffirmed [15, 16]. However, a lack of region or nation-wise estimation hinders tracking the trends and progress relating to LBW status.

The etiology of LBW is known to be multifactorial. Different studies have highlighted several contributing factors for LBW, including genetic, nutritional, demographic, and psychosocial factors, preterm birth, maternal morbidity during pregnancy, care during pregnancy, obstetric complication, antenatal care, multiple pregnancies, etc. [4, 17, 18]. The prevention and necessary intervention of LBW is of utmost priority from the public health lens.

Studies found about 4% decline in LBW from NFHS-3 to NFHS-4 due to a significant improvement of maternal health in India since 2005 by national-level health programs, but no such improvement of LBW was found between NFHS-4 and NFHS-5 [12, 19–21]. According to the findings of a study on NFHS-4, LBW was influenced by a low family wealth index, lower maternal education, and delivery at home, emphasising the need for region-specific identification of linked factors responsible for LBW and strategies to address them [22, 23]. Hence, it is crucial to investigate what the region-specific contributing factors are for the lack of progress in LBW between NFHS-4 and NFHS-5.

The focus of this study is to address the prevailing informational gap region-wise, revealed by the most recent NFHS-5 (2019 – 2021) data. The findings will have tremendous policy implications in terms of the nature of intervention to be carried out in different regions. The present study was carried out to find the burden and determinants of LBW based on India’s regional and residential (rural–urban) divisions.

## Methods

### Study design and subject

The unit-level data for this study were obtained from a cross-sectional survey designated the National Family Health Survey of 2019–21 (NFHS-5). This NFHS-5 was a national household survey conducted in India by International Institute for Population Sciences (IIPS) that adhered to the DHS's guiding principles by using standardised questionnaires, sample strategies, and field methodology [20]. A total of 209,223 births of children under the age of five were included in the statistical analysis.

### Inclusion and exclusion criteria

**Fig. 1:**
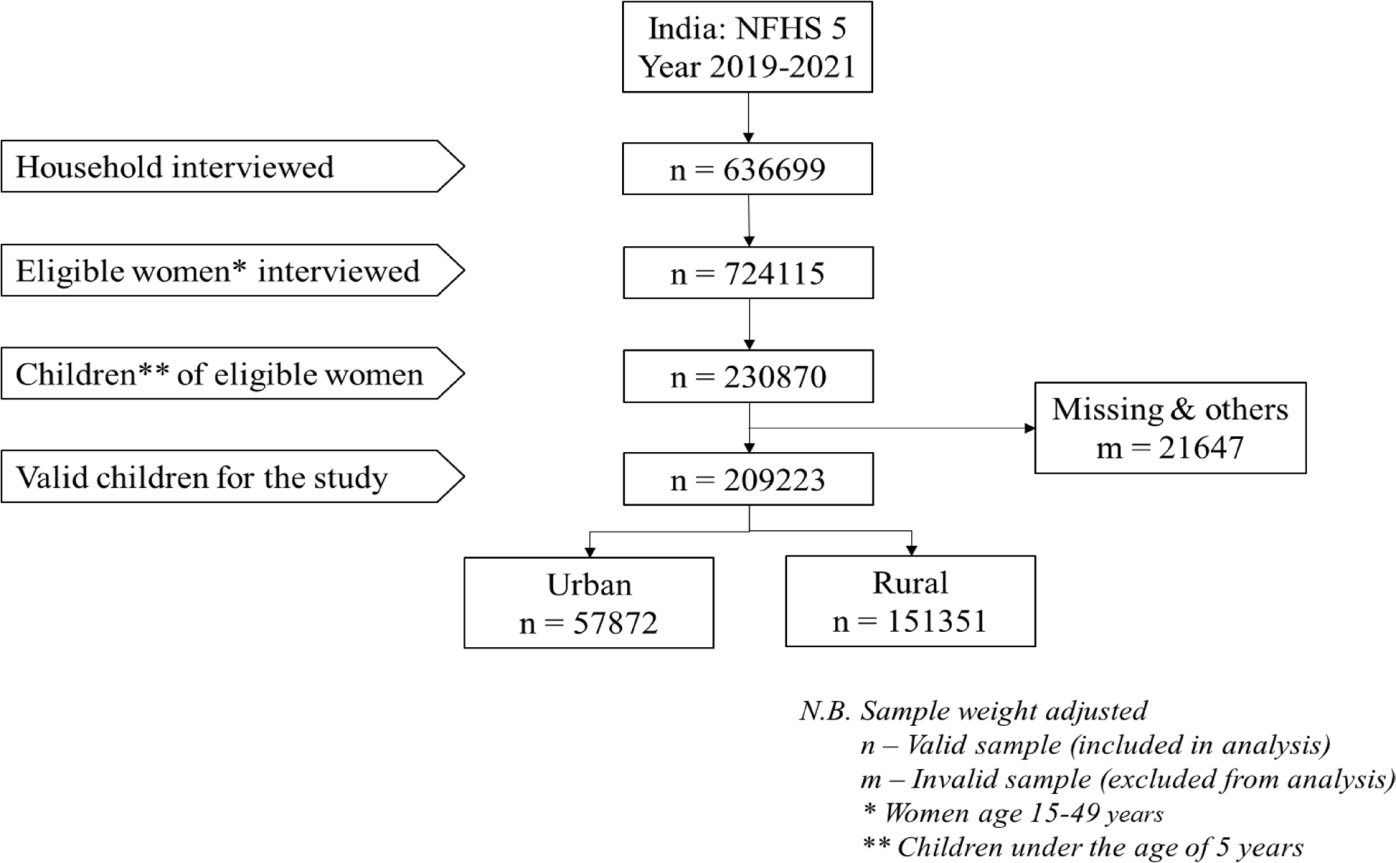
Sample selection of under-five children for the statistical analysis

This study included only Indian women currently residing in India and was of reproductive age (15–49 years). Among the chosen women, children under the age of five were considered for this study. The study excluded women and their children whose sociodemographic or anthropometric data were either missing or incomplete. The sample selection process for the study was shown in the flow chart (Fig. 1).

### Unit-level study variables

#### Outcome variable

The outcome variable was low birth weight of the under-five children; it was measured by their birth weight (BW), if BW < 2500 g was considered as LBW, and if BW ≥ 2500 g was considered normal [24, 25].

#### Independent variables

These variables were divided into two categories: grouping factors and explanatory factors. The six regional divisions of India based on NFHS have been considered as grouping factors and the entire study has been conducted based on these zonal distinctions. Socioeconomic, demographic, and maternal characteristics were included in the explanatory factors. Most of the explanatory factors were based on prior studies and were available in the NFHS-5 dataset.

#### Operational definition and classifications

The wealth index measured a household's wealth based on the quantity and variety of consumer goods it owns, its assets, and housing characteristics, including its access to drinking water, toilet facilities, and flooring [20]. The distribution was split into five equal groups, with the richest families receiving the highest score and the poorest receiving the lowest. Full antenatal care was defined as four or more antenatal visits, at least one tetanus toxoid (TT) injection, and confirmed consumption of iron folic acid (IFA) tablets or syrup for at least 100 days [12, 26]. Body mass index (BMI) was calculated by dividing weight in kilogrammes by height in metres squared (kg/m^2^). According to the WHO (2004), BMI was classified as underweight if it was < 18.5; normal was considered to be between ≥ 18 and < 25; and overweight was ≥ 25 [27]. A woman was considered anaemic when her haemoglobin level was < 11 g per decilitre (g/dl) for pregnant women [20]. The social category as specified by the Constitution was determined by whether the head of the household self-identified as being a member of a scheduled caste (SC), scheduled tribe (ST), other backward class (OBC), except for these three, were grouped under the general category [28, 29].

#### Statistical analysis

The summary of overall India and urban–rural variation of the variables were defined using frequencies and percentages for qualitative data. The differences in the proportional frequency of LBW between urban and rural were analysed using the Z-proportion test. The Chi-square (χ^2^) test was used to determine the association between low birth weight and socioeconomic, demographic, and maternal characteristics regarding the urban–rural of residential settings. The variables found to be statistically significant by the χ^2^-test were included in binary logistic models as independent variables. Independent variables were further examined for multicollinearity issues using the variance inflation factor (VIF), which was considered less than 5. Statistical significance was estimated using a 95% confidence interval (CI), and p ≤ 0.05 was considered significant. The logistic regression model was validated to see if the data fit correctly using the Omnibus Chi-square test [30, 31]. Individual effect of the explanatory variable was determined using crude odds ratio (COR), and the cumulative effect of the adjusting explanatory variables were determined using adjusted odds ratio (AOR). The model’s accuracy was tested by the sensitivity and specificity of the ‘predicted probability’ values and receiver operator characteristic (ROC) curve [32]. The Youden index (*J*) was applied to the ROC curve to assess the efficiency of explanatory variables in LBW. It is defined as Youden's *J* = sensitivity + specificity − 1. Higher *J* -values in the evaluations would be preferable [33]. All statistical analyses were carried out using Microsoft Excel and SPSS (version IBM 25).

## Results

**Fig. 2:**
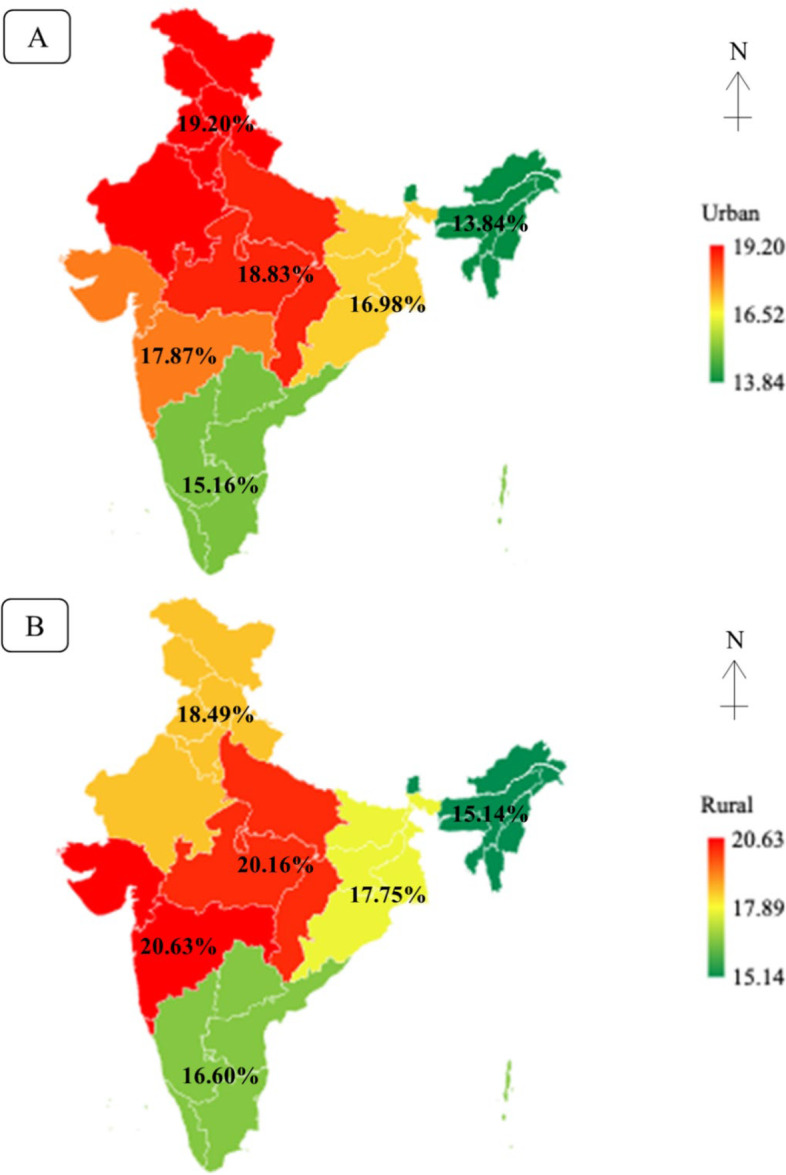
Rate map showing distribution of LBW by the region based on NFHS-5. **A** Urban and (**B**) Rural

Out of the total births of 209,223, about 18.24% of the children were born with LBW in India. The proportion of newborn having LBW was found to be significantly higher in rural areas (18.58%) as compared to the urban areas (17.36%) of India (Z-value = 2.72, *p* = 0.007). Additionally, this study estimated that the prevalence of LBW in newborns was more prevalent in western (20.63%) and central (20.16%) rural areas. In contrast, the majority of LBW was comparatively higher in northern (19.20%) and central (18.83%) urban areas (Fig. 2).

### Distribution of independent variables based on LBW

Table 1 shows that the frequency of all explanatory factors including sociodemographic and maternal factors were significantly (χ^2^-value, *p* < 0.05) associated with the birth weight, irrespective of their place of residence i.e. urban and rural. Among the sociodemographic factors in urban area, the occurrence of LBW was found to be significantly higher among the Hindus (17.93%), Schedule Caste group (18.74%), 4th or higher birth order (20.53%), pregnancies delivered at home (20.83%), larger families with more than four persons (17.59%) and poor wealth indexed households (21.01%). Contrarily, sociodemographic factors in rural areas were found to have a higher the occurrence of LBW among the other religious groups (21.00%), Schedule castes (19.79%), 1st child (19.56%), home birth deliveries (21.68%), smaller families with less or equal of 4 persons (19.52%) and households under poor wealth index (20.18%).

**Table 1:** Distribution of independent variables based on LBW in urban and rural India

**Independent Variables**	Low Birth Weight (< 2500gm)		
	India % (n)	Urban % (n)	Rural % (n)
***Grouping factor***			
Region			
Central	19.88 (11,181)	18.83 (2251)	20.16 (8929)
East	17.61 (9027)	16.98 (1587)	17.75 (7441)
North	18.71 (5415)	19.20 (1760)	18.49 (3656)
Northeast	14.93 (1121)	13.84 (160)	15.14 (962)
South	16.04 (6026)	15.16 (2238)	16.60 (3788)
West	19.48 (5397)	17.87 (2052)	20.63 (3345)
χ^2^-value [*p-*value]	326.12 [< 0.001]	102.28 [< 0.001]	246.47 [< 0.001]
***Explanatory factors***			
Religion			
Hindu	18.55 (30,987)	17.93 (7672)	18.76 (23,315)
Muslim	16.83 (5534)	15.88 (1963)	17.40 (3571)
Christian	15.85 (691)	15.11 (199)	16.17 (492)
Other^a^	19.35 (955)	15.21 (213)	21.00 (742)
χ^2^-value [*p-*value]	75.18 [< 0.001]	37.48 [< 0.001]	46.86 [< 0.001]
Social category			
Schedule tribe	18.76 (3893)	14.71 (362)	19.30 (3531)
Schedule caste	19.54 (9328)	18.74 (2146)	19.79 (7182)
OBC	17.79 (16,171)	17.30 (4369)	17.98 (11,802)
General	17.32 (6627)	16.34 (2477)	17.96 (4151)
χ^2^-value [*p-*value]	91.90 [< 0.001]	37.80 [< 0.001]	62.89 [< 0.001]
Birth order			
1st	18.92 (16,090)	17.44 (4442)	19.56 (11,649)
2nd	17.50 (12,531)	16.61 (3515)	17.87 (9017)
3rd	17.75 (5470)	17.54 (1270)	17.82 (4199)
≥ 4th	18.73 (4075)	20.53 (820)	18.32 (3255)
χ^2^-value [*p-*value]	61.45 [< 0.001]	36.55 [< 0.001]	64.33 [< 0.001]
Place of delivery			
Home	21.56 (2660)	20.83 (370)	21.68 (2290)
Institution	18.03 (35,430)	17.23 (9648)	18.35 (25,782)
χ^2^-value [*p-*value]	96.75 [< 0.001]	15.58 [< 0.001]	71.83 [< 0.001]
Family size			
≤ 4	18.65 (10,176)	16.83 (2970)	19.52 (7206)
> 4	18.10 (27,990)	17.59 (7077)	18.28 (20,914)
χ^2^-value [*p-*value]	8.30 [0.004]	4.97 [0.026]	28.65 [< 0.001]
Wealth Index			
Poor	20.25 (18,463)	21.01 (1505)	20.18 (16,958)
Middle	17.47 (7378)	18.48 (1817)	17.17 (5561)
Rich	16.26 (12,325)	16.45 (6725)	16.04 (5601)
χ^2^-value [*p-*value]	461.96 [< 0.001]	98.72 [< 0.001]	334.25 [< 0.001]
Mother's age group (years)			
15–24	19.84 (13,818)	18.11 (2721)	20.31 (11,096)
25–34	17.44 (21,453)	17.07 (6340)	17.60 (15,113)
35–49	17.47 (2896)	17.27 (986)	17.58 (1910)
χ^2^-value [*p-*value]	177.99 [< 0.001]	8.07 [0.018]	169.40 [< 0.001]
Intended pregnancy			
Wanted then	18.03 (34,446)	17.08 (9094)	18.39 (25,352)
Wanted later	21.13 (2093)	21.88 (567)	20.87 (1526)
Wanted no more	19.78 (1628)	19.01 (386)	20.04 (1242)
χ^2^-value [*p-*value]	74.54 [< 0.001]	43.61 [< 0.001]	37.17 [< 0.001]
Mother's education			
No education	20.10 (7824)	20.19 (1162)	20.08 (6663)
Primary	20.63 (5125)	22.98 (1181)	20.02 (3944)
Secondary	18.24 (20,112)	17.46 (5246)	18.54 (14,866)
Higher	14.50 (5104)	14.52 (2458)	14.49 (2647)
χ^2^-value [*p-*value]	515.25 [< 0.001]	240.79 [< 0.001]	278.73 [< 0.001]
Received full ANC			
Yes	16.09 (8590)	15.30 (3031)	16.56 (5560)
No	18.54 (19,999)	17.93 (4852)	18.74 (15,147)
χ^2^-value [*p-*value]	146.35 [< 0.001]	56.50 [< 0.001]	75.91 [< 0.001]
Timing of first ANC visit			
1st trimester	17.25 (19,946)	16.27 (5832)	17.69 (14,114)
2nd trimester	18.44 (5456)	18.28 (1237)	18.49 (4219)
3rd trimester	18.69 (1479)	18.18 (454)	18.91 (1024)
χ^2^-value [*p-*value]	30.53 [< 0.001]	20.83 [< 0.001]	11.51 [0.003]
Mother's BMI (kg/m^2^)			
Underweight	22.00 (8467)	21.55 (1428)	22.09 (7040)
Normal	17.75 (21,919)	17.08 (5327)	17.97 (16,593)
Overweight & Obese	16.09 (6445)	16.34 (2741)	15.91 (3705)
χ^2^-value [*p-*value]	508.40 [< 0.001]	94.64 [< 0.001]	391.95 [< 0.001]
Mother's Anaemia			
Non anaemic	17.69 (14,331)	17.00 (4134)	17.99 (10,197)
Anaemic	18.60 (22,088)	17.69 (5205)	18.90 (16,883)
χ^2^-value [*p-*value]	26.35 [< 0.001]	4.39 [0.036]	18.80 [< 0.001]

^a^*Sikh, Buddhist/Neo-Buddhist, Jain, Jewish, Parsi/Zoroastrian, No religion, and Other*

The results also show that among the maternal factors in urban areas, the prevalence of LBW was considerably higher among mothers aged 15–24 years (18.11%), unintended pregnancies those were wanted later (21.88%), who had attended up to a primary level of education (20.19%), had not received full ANC (17.93%), had only received ANC during the 2nd trimester (18.28%), was underweight (21.55%), and was anaemic (17.69%). Rural areas have shown an almost similar trend in maternal factors, in contrast to urban areas, unlike the factors such as mothers with no formal education (20.08%) and mother who received ANC in the 3^rd^ trimester had a higher prevalence of LBW.

### Factors affecting LBW

Tables 2 & 4 shows crude (COR) and 2B & 3B shows adjusted (AOR) contribution of explanatory factors on LBW by regional divisions using binary logistic regression. The omnibus chi-square for urban (Table 3) and rural (Table 5) areas was significant (*p* < 0.05) in all regional divisions, including India. The correct percentage of prediction of the adjusted model for India, Central, East, North, Northeast, South and West in the urban areas 83.38%, 81.48%, 83.84%, 81.83%, 86.96%, 85.96% and 82.49%, while in the rural areas 82.09%, 80.49%, 82.43%, 82.44%, 85.85%, 84.35% and 80.62%, respectively.

**Table 2:** Crude odds ratio for effects of sociodemographic and maternal factors on low birth weight in Urban India using binary logistic regression

		Regions												
Explanatory Factors	India		Central		East		North		Northeast		South		West	
	COR (95% CI)	*p-*value	COR (95% CI)	*p-*value	COR (95% CI)	*p-*value	COR (95% CI)	*p-*value	COR (95% CI)	*p-*value	COR (95% CI)	*p-*value	COR (95% CI)	*p-*value
Religion (Hindu ®)														
Muslim	0.86 (0.82, 0.91)	< 0.001	0.93 (0.83, 1.04)	0.178	0.90 (0.79, 1.03)	0.122	0.78 (0.67, 0.90)	0.001	1.05 (0.68, 1.62)	0.822	0.94 (0.83, 1.05)	0.258	0.76 (0.67, 0.85)	< 0.001
Christian	0.81 (0.70, 0.95)	0.008	1.66 (0.75, 3.68)	0.212	0.50 (0.23, 1.09)	0.081	0.98 (0.36, 2.69)	0.970	0.35 (0.20, 0.61)	< 0.001	0.94 (0.77, 1.15)	0.550	2.53 (1.73, 3.70)	< 0.001
Other	0.82 (0.71, 0.95)	0.009	0.80 (0.44, 1.46)	0.469	0.61 (0.23, 1.64)	0.326	0.84 (0.68, 1.05)	0.121	0.57 (0.20, 1.61)	0.288	1.96 (1.09, 3.52)	0.026	0.60 (0.46, 0.77)	< 0.001
Social category (General ®)														
Schedule tribe	0.88 (0.78, 1.00)	0.043	0.89 (0.68, 1.17)	0.412	1.12 (0.83, 1.50)	0.464	1.61 (1.12, 2.31)	0.010	0.50 (0.28, 0.90)	0.021	1.04 (0.79, 1.37)	0.788	0.86 (0.68, 1.09)	0.212
Schedule caste	1.18 (1.11, 1.26)	< 0.001	1.11 (0.97, 1.28)	0.129	1.46 (1.26, 1.69)	< 0.001	1.30 (1.14, 1.48)	< 0.001	1.41 (0.83, 2.42)	0.206	1.12 (0.93, 1.34)	0.234	1.08 (0.93, 1.25)	0.341
OBC	1.07 (1.01, 1.13)	0.013	1.03 (0.92, 1.16)	0.626	1.00 (0.87, 1.15)	0.999	1.06 (0.93, 1.21)	0.380	1.31 (0.77, 2.23)	0.312	1.26 (1.07, 1.47)	0.004	1.25 (1.12, 1.41)	< 0.001
Birth order (1st ®)														
2nd	0.94 (0.90, 0.99)	0.018	0.91 (0.82, 1.01)	0.081	1.00 (0.88, 1.13)	0.980	1.04 (0.93, 1.18)	0.471	0.69 (0.47, 1.02)	0.061	0.85 (0.77, 0.93)	0.001	1.01 (0.91, 1.13)	0.826
3rd	1.01 (0.94, 1.08)	0.835	0.87 (0.76, 1.00)	0.054	1.04 (0.88, 1.24)	0.625	1.29 (1.10, 1.52)	0.002	0.71 (0.38, 1.29)	0.260	0.94 (0.81, 1.10)	0.455	0.95 (0.81, 1.11)	0.505
≥ 4th	1.22 (1.13, 1.33)	< 0.001	1.08 (0.93, 1.26)	0.329	0.92 (0.75, 1.13)	0.444	1.54 (1.27, 1.87)	< 0.001	0.84 (0.43, 1.61)	0.592	1.09 (0.84, 1.42)	0.509	1.46 (1.22, 1.74)	< 0.001
Place of delivery (Institution ®)														
Home	1.27 (1.13, 1.42)	< 0.001	1.15 (0.96, 1.39)	0.137	0.85 (0.65, 1.12)	0.247	1.59 (1.22, 2.07)	0.001	0.72 (0.29, 1.79)	0.479	1.85 (1.21, 2.84)	0.005	1.47 (1.07, 2.03)	0.017
Family size (≤ 4 ®)														
> 4	1.05 (1.01, 1.11)	0.026	0.92 (0.83, 1.03)	0.138	0.94 (0.84, 1.06)	0.327	1.07 (0.95, 1.21)	0.256	0.97 (0.69, 1.36)	0.854	1.07 (0.98, 1.18)	0.136	1.09 (0.98, 1.21)	0.133
Wealth Index (Rich ®)														
Poor	1.35 (1.27, 1.44)	< 0.001	1.41 (1.24, 1.60)	< 0.001	1.55 (1.37, 1.75)	< 0.001	1.41 (1.13, 1.76)	0.002	1.68 (1.13, 2.51)	0.010	1.52 (1.29, 1.79)	< 0.001	1.01 (0.85, 1.20)	0.899
Middle	1.15 (1.09, 1.22)	< 0.001	1.03 (0.91, 1.17)	0.660	1.17 (1.02, 1.35)	0.023	1.45 (1.25, 1.68)	< 0.001	1.27 (0.84, 1.93)	0.257	1.05 (0.93, 1.19)	0.400	1.41 (1.25, 1.60)	< 0.001
Mother's age group (35–49 ®)														
15–24 yrs	1.06 (0.98, 1.15)	0.157	1.26 (1.06, 1.50)	0.008	1.12 (0.90, 1.40)	0.303	1.07 (0.88, 1.30)	0.497	1.32 (0.78, 2.24)	0.304	0.79 (0.67, 0.93)	0.006	1.18 (0.99, 1.42)	0.069
25–34 yrs	0.99 (0.92, 1.06)	0.716	1.10 (0.94, 1.28)	0.247	0.98 (0.79, 1.21)	0.818	0.91 (0.76, 1.08)	0.264	1.17 (0.73, 1.88)	0.513	0.80 (0.69, 0.93)	0.004	1.17 (0.99, 1.38)	0.071
Intended pregnancy (Wanted then ®)														
Wanted later	1.36 (1.23, 1.50)	< 0.001	1.23 (1.00, 1.50)	0.045	1.53 (1.25, 1.86)	< 0.001	1.62 (1.34, 1.96)	< 0.001	0.99 (0.45, 2.21)	0.988	0.68 (0.49, 0.95)	0.022	1.44 (1.12, 1.85)	0.004
Wanted no more	1.14 (1.02, 1.28)	0.023	1.00 (0.79, 1.27)	0.972	0.73 (0.55, 0.98)	0.033	1.34 (1.08, 1.67)	0.008	1.16 (0.48, 2.78)	0.737	1.35 (1.00, 1.81)	0.047	1.31 (0.99, 1.72)	0.060
Mother's education (Higher ®)														
No education	1.49 (1.38, 1.61)	< 0.001	1.43 (1.24, 1.66)	< 0.001	1.47 (1.23, 1.76)	< 0.001	1.72 (1.45, 2.04)	< 0.001	1.18 (0.57, 2.44)	0.650	1.36 (1.08, 1.70)	0.008	1.05 (0.85, 1.30)	0.644
Primary	1.76 (1.63, 1.90)	< 0.001	1.80 (1.53, 2.13)	< 0.001	1.58 (1.31, 1.89)	< 0.001	1.80 (1.50, 2.15)	< 0.001	1.16 (0.65, 2.09)	0.613	1.76 (1.44, 2.15)	< 0.001	1.61 (1.35, 1.91)	< 0.001
Secondary	1.25 (1.18, 1.31)	< 0.001	1.40 (1.25, 1.57)	< 0.001	0.92 (0.80, 1.07)	0.276	1.43 (1.26, 1.63)	< 0.001	0.81 (0.54, 1.23)	0.320	1.30 (1.18, 1.44)	< 0.001	1.16 (1.03, 1.30)	0.013
Received full ANC (Yes ®)														
No	1.21 (1.15, 1.27)	< 0.001	1.15 (1.02, 1.30)	0.021	1.11 (0.98, 1.26)	0.105	1.21 (1.07, 1.36)	0.002	1.29 (0.89, 1.89)	0.181	1.09 (0.98, 1.21)	0.116	1.26 (1.13, 1.40)	< 0.001
Timing of first ANC visit (1st trimester ®)														
2nd trimester	1.15 (1.08, 1.23)	< 0.001	0.94 (0.82, 1.08)	0.372	1.34 (1.15, 1.56)	< 0.001	1.28 (1.09, 1.51)	0.003	1.26 (0.81, 1.97)	0.308	0.86 (0.71, 1.03)	0.108	1.28 (1.10, 1.48)	0.001
3rd trimester	1.14 (1.03, 1.27)	0.012	1.40 (1.10, 1.78)	0.007	1.07 (0.78, 1.46)	0.672	1.68 (1.26, 2.24)	< 0.001	0.63 (0.20, 1.98)	0.428	0.86 (0.70, 1.06)	0.163	1.31 (1.07, 1.61)	0.008
Mother's BMI (Normal ®)														
Underweight	1.33 (1.25, 1.42)	< 0.001	1.31 (1.13, 1.51)	< 0.001	1.46 (1.26, 1.69)	< 0.001	1.44 (1.21, 1.72)	< 0.001	1.91 (1.22, 3.00)	0.005	1.43 (1.24, 1.66)	< 0.001	1.15 (1.00, 1.33)	0.043
Overweight & Obese	0.95 (0.90, 1.00)	0.038	0.84 (0.75, 0.94)	0.002	0.85 (0.74, 0.97)	0.020	0.97 (0.86, 1.10)	0.650	0.67 (0.42, 1.07)	0.093	1.07 (0.97, 1.18)	0.170	1.08 (0.96, 1.20)	0.199
Mother's Anemia (Non anemic ®)														
Anemic	1.05 (1.00, 1.10)	0.036	1.17 (1.06, 1.29)	0.002	1.02 (0.90, 1.14)	0.802	1.07 (0.96, 1.19)	0.242	1.74 (1.21, 2.50)	0.003	0.99 (0.90, 1.09)	0.804	0.92 (0.83, 1.02)	0.104

*®* = *Reference category*

**Table 3:** Adjusted odds ratio for effects of sociodemographic and maternal factors on low birth weight in Urban India using binary logistic regression

			Regions											
Explanatory Factors	India		Central		East		North		Northeast		South		West	
	AOR (95% CI)	*p-*value	AOR (95% CI)	*p-*value	AOR (95% CI)	*p-*value	AOR (95% CI)	*p-*value	AOR (95% CI)	*p-*value	AOR (95% CI)	*p-*value	AOR (95% CI)	*p-*value
Religion (Hindu ®)														
Muslim	0.76 (0.70, 0.82)	< 0.001	0.96 (0.82, 1.12)	0.578	0.77 (0.62, 0.94)	0.011	0.68 (0.55, 0.84)	< 0.001	0.75 (0.25, 2.23)	0.603	0.87 (0.74, 1.02)	0.085	0.58 (0.49, 0.69)	< 0.001
Christian	1.01 (0.84, 1.22)	0.906	4.30 (1.61, 11.53)	0.004	0.63 (0.26, 1.56)	0.319	0.95 (0.24, 3.67)	0.934	0.44 (0.16, 1.24)	0.121	1.17 (0.92, 1.50)	0.193	4.37 (2.68, 7.12)	< 0.001
Other	0.87 (0.72, 1.05)	0.140	0.55 (0.21, 1.47)	0.231	0.47 (0.12, 1.88)	0.287	0.76 (0.58, 1.01)	0.055	1.00 (0.29, 3.49)	0.998	5.41 (2.13, 13.78)	< 0.001	0.74 (0.52, 1.04)	0.082
Social category (General ®)														
Schedule tribe	0.79 (0.68, 0.91)	0.001	0.75 (0.54, 1.04)	0.085	0.90 (0.62, 1.31)	0.586	1.25 (0.80, 1.96)	0.331	0.88 (0.35, 2.18)	0.777	0.83 (0.59, 1.17)	0.286	0.95 (0.72, 1.25)	0.714
Schedule caste	1.01 (0.93, 1.09)	0.851	0.94 (0.78, 1.12)	0.482	1.31 (1.08, 1.59)	0.007	1.08 (0.91, 1.28)	0.368	1.18 (0.61, 2.28)	0.615	0.94 (0.74, 1.18)	0.570	0.92 (0.76, 1.11)	0.378
OBC	1.02 (0.95, 1.08)	0.661	0.89 (0.77, 1.02)	0.102	1.09 (0.91, 1.30)	0.349	1.00 (0.86, 1.18)	0.961	1.04 (0.57, 1.90)	0.905	1.22 (1.00, 1.47)	0.047	1.12 (0.97, 1.29)	0.123
Birth order (1st ®)														
2nd	0.90 (0.84, 0.96)	0.001	0.88 (0.76, 1.02)	0.086	0.86 (0.72, 1.01)	0.072	1.04 (0.89, 1.22)	0.600	0.74 (0.45, 1.24)	0.256	0.91 (0.80, 1.03)	0.131	0.83 (0.72, 0.96)	0.011
3rd	0.86 (0.79, 0.95)	0.003	0.87 (0.72, 1.06)	0.179	0.79 (0.61, 1.03)	0.079	1.04 (0.83, 1.31)	0.726	0.57 (0.21, 1.55)	0.267	0.87 (0.71, 1.07)	0.194	0.73 (0.58, 0.92)	0.007
≥ 4th	1.02 (0.90, 1.15)	0.805	1.08 (0.85, 1.37)	0.540	0.66 (0.47, 0.92)	0.014	1.48 (1.11, 1.98)	0.007	0.67 (0.19, 2.41)	0.539	0.72 (0.49, 1.05)	0.089	1.25 (0.95, 1.66)	0.114
Place of delivery (Institution ®)														
Home	1.16 (0.99, 1.36)	0.059	1.14 (0.90, 1.46)	0.282	0.99 (0.68, 1.43)	0.951	1.09 (0.75, 1.60)	0.647	0.87 (0.23, 3.35)	0.844	1.63 (0.87, 3.08)	0.130	1.94 (1.27, 2.95)	0.002
Family size (≤ 4 ®)														
> 4	1.04 (0.98, 1.11)	0.189	0.91 (0.80, 1.05)	0.195	1.09 (0.93, 1.27)	0.288	1.10 (0.94, 1.29)	0.229	0.93 (0.58, 1.49)	0.751	1.05 (0.93, 1.18)	0.468	0.98 (0.85, 1.13)	0.802
Wealth Index (Rich ®)														
Poor	1.28 (1.17, 1.40)	< 0.001	1.38 (1.15, 1.64)	< 0.001	1.58 (1.30, 1.93)	< 0.001	1.11 (0.81, 1.53)	0.514	1.21 (0.62, 2.34)	0.578	1.46 (1.17, 1.82)	0.001	1.56 (1.22, 2.00)	< 0.001
Middle	1.03 (0.95, 1.11)	0.485	0.84 (0.71, 0.99)	0.042	1.24 (1.03, 1.51)	0.025	1.30 (1.05, 1.60)	0.016	1.06 (0.59, 1.89)	0.853	1.03 (0.88, 1.20)	0.720	1.43 (1.20, 1.70)	< 0.001
Mother's age group (35–49 ®)														
15–24 yrs	1.00 (0.90, 1.12)	0.991	1.28 (1.00, 1.63)	0.054	0.80 (0.60, 1.08)	0.153	0.99 (0.76, 1.30)	0.949	0.88 (0.38, 2.02)	0.758	0.86 (0.70, 1.07)	0.178	1.18 (0.91, 1.53)	0.202
25–34 yrs	0.96 (0.88, 1.06)	0.435	1.16 (0.95, 1.42)	0.156	0.76 (0.59, 1.00)	0.046	0.94 (0.75, 1.17)	0.565	1.13 (0.58, 2.20)	0.714	0.79 (0.66, 0.95)	0.012	1.15 (0.92, 1.43)	0.230
Intended pregnancy (Wanted then ®)														
Wanted later	1.41 (1.24, 1.60)	< 0.001	1.73 (1.35, 2.21)	< 0.001	1.09 (0.82, 1.46)	0.540	1.80 (1.40, 2.31)	< 0.001	1.40 (0.51, 3.81)	0.515	0.57 (0.35, 0.91)	0.018	1.33 (0.91, 1.94)	0.138
Wanted no more	1.14 (0.98, 1.33)	0.084	1.02 (0.74, 1.39)	0.915	0.59 (0.39, 0.90)	0.014	1.11 (0.82, 1.51)	0.511	0.82 (0.14, 4.89)	0.824	1.31 (0.89, 1.93)	0.174	2.62 (1.83, 3.74)	< 0.001
Mother's education (Higher ®)														
No education	1.28 (1.14, 1.44)	< 0.001	1.26 (1.01, 1.57)	0.039	1.29 (0.96, 1.74)	0.090	1.26 (0.96, 1.65)	0.095	0.92 (0.25, 3.41)	0.899	1.02 (0.74, 1.42)	0.890	0.73 (0.53, 1.02)	0.063
Primary	1.80 (1.62, 2.01)	< 0.001	1.94 (1.56, 2.42)	< 0.001	1.56 (1.18, 2.05)	0.002	1.32 (1.01, 1.72)	0.041	1.04 (0.42, 2.59)	0.929	2.06 (1.59, 2.68)	< 0.001	1.51 (1.18, 1.94)	0.001
Secondary	1.26 (1.18, 1.35)	< 0.001	1.39 (1.20, 1.62)	< 0.001	0.82 (0.67, 1.00)	0.053	1.38 (1.16, 1.63)	< 0.001	0.67 (0.37, 1.21)	0.181	1.39 (1.22, 1.59)	< 0.001	1.17 (1.00, 1.37)	0.047
Received full ANC (Yes ®)														
No	1.13 (1.07, 1.19)	< 0.001	1.05 (0.92, 1.19)	0.516	1.03 (0.88, 1.19)	0.724	1.09 (0.95, 1.24)	0.230	1.02 (0.64, 1.62)	0.941	1.07 (0.95, 1.20)	0.276	1.25 (1.11, 1.42)	< 0.001
Timing of first ANC visit (1st trimester ®)														
2nd trimester	1.09 (1.01, 1.17)	0.031	0.90 (0.78, 1.05)	0.171	1.32 (1.11, 1.57)	0.001	1.17 (0.97, 1.40)	0.098	1.02 (0.59, 1.79)	0.933	0.87 (0.71, 1.07)	0.185	1.26 (1.07, 1.49)	0.006
3rd trimester	1.13 (1.00, 1.27)	0.048	1.34 (1.03, 1.73)	0.030	1.00 (0.69, 1.45)	0.988	1.69 (1.23, 2.32)	0.001	0.17 (0.02, 1.84)	0.144	0.89 (0.71, 1.11)	0.312	1.18 (0.92, 1.50)	0.196
Mother's BMI (Normal ®)														
Underweight	1.36 (1.26, 1.47)	< 0.001	1.35 (1.13, 1.60)	0.001	1.56 (1.30, 1.89)	< 0.001	1.44 (1.16, 1.79)	0.001	1.98 (1.09, 3.60)	0.025	1.48 (1.24, 1.77)	< 0.001	1.09 (0.92, 1.29)	0.341
Overweight & Obese	1.02 (0.96, 1.08)	0.551	0.92 (0.80, 1.05)	0.213	0.99 (0.83, 1.18)	0.871	1.12 (0.96, 1.29)	0.151	0.85 (0.49, 1.48)	0.561	1.00 (0.89, 1.13)	0.972	1.19 (1.03, 1.37)	0.015
Mother's Anemia (Non anemic ®)														
Anemic	0.99 (0.94, 1.05)	0.777	1.10 (0.98, 1.24)	0.101	0.90 (0.78, 1.05)	0.166	1.07 (0.94, 1.22)	0.337	1.82 (1.12, 2.93)	0.015	0.90 (0.80, 1.00)	0.057	0.97 (0.86, 1.10)	0.668

*®* = *Reference category*

#### Urban India

Tables 2 and 3 shows the effect of selected sociodemographic and maternal factors on LBW in and across different urban regions of India. In and across different regions of urban India, religion, social category, birth order, wealth Index, intended pregnancy, mother's education, received full ANC, timing of first ANC visit, and mother's BMI were common significant factors for both crude and adjusted odds that affect LBW. The timing of first ANC visit had showed significant impact only in urban areas while considering crude and adjusted odds.

Concerning the religion and social categories in urban areas of urban India, children in religious Muslims (COR = 0.86, CI: 0.82–0.91, *p* < 0.001; AOR = 0.76, CI: 0.70–0.82, *p* < 0.001) and STs (COR = 0.88, CI: 0.78–1.00, *p* = 0.043; AOR = 0.79, CI: 0.68–0.91, *p* = 0.001) had a significantly lower risk of LBW than Hindus and General social category, respectively. Furthermore, it appears in the cumulative effect based on AOR that Christians were over four times more likely to suffer from LBW in the urban area of central and western India, while Muslim children were less likely to experience it in the eastern, northern, and western regions, and other religious groups experienced it more likely (over five times) than Hindu children in the southern region. Compared to children from general castes, SCs in east (COR = 1.46, CI: 1.26–1.69, *p* < 0.001; AOR = 1.31, CI: 1.08–1.59, *p* = 0.007) and OBCs in south (COR = 1.26, CI: 1.07–1.47, *p* = 0.004; AOR = 1.22, CI: 1.00–1.47, *p* = 0.047) were more likely to be suffered from LBW. The probability of LBW was significantly reduced in the 2nd and 3rd child in urban India including west region, and more or equal of 4th children in the east region based on AOR. In urban western India, children who born at home were more likely to have LBW (COR = 1.47, CI: 1.07–2.03, *p* = 0.017; AOR = 1.94, CI: 1.27–2.95, *p* = 0.002) than the healthcare institutions. In both unadjusted and adjusted conditions, compared to mothers from the rich wealth indexed family, the lower category (poor and middle class) were more likely to have LBW children in the central, east, north, south and west regions.

Regarding maternal concerns, in the southern regions of India, mothers aged 25–34 were less likely to have LBW children (COR = 0.80, CI: 0.69–0.93, *p* = 0.004; AOR = 0.79, CI: 0.66–0.95, *p* = 0.012) than 35–49 years. It was found that the risk of LBW was more likely among the unintended pregnancies in almost all regions except for eastern and northeastern part for both COR and AOR. Mother’s educational status was one of the most influential factors for LBW in urban area. Compared to higher educated mothers in central, east, north, south, and west regions of India the lower educated mothers were more likely to have LBW children based on COR and AOR. Also LBW was more prevalent among children whose mothers did not receive full ANC in urban India (COR = 1.21, CI: 1.15–1.27, *p* < 0.001; AOR = 1.13, CI: 1.07–1.19, *p* < 0.001) and western region (COR = 1.26, CI: 1.13–1.40, *p* < 0.001; AOR = 1.25, CI: 1.11–1.42, *p* < 0.001). Based on COR and AOR, the prevalence of LBW was also enhanced in the central, eastern, northern, and western regions by receiving the first ANC visit after the 1st trimester. Maternal underweight was found to be a common risk factor for LBW in all regions of India both for COR and AOR, with the highest prevalence occurring in the northeastern region (COR = 1.91, CI: 1.22–3.00, *p* = 0.005; AOR = 1.98, CI: 1.09–3.60, *p* = 0.025). Anaemic mothers were more likely to have LBW children in the northeast region (COR = 1.74, CI: 1.21–2.50, *p* = 0.003; AOR = 1.82, CI: 1.12–2.93, *p* = 0.015). The place of delivery and mother’s anemia had significant individual impact on LBW in urban India but became non-significant when adjusted for other variables.

#### Rural India

Similar to urban areas, Tables 4 and 5 exhibits how certain sociodemographic and maternal factors affect LBW in rural India. In and across different regions of rural India, religion, social category, birth order, place of delivery, family size, wealth Index, mother's age group, intended pregnancy, mother's education, received full ANC, and mother's BMI were common significant factors for both crude and adjusted odds that affect LBW. The factors such as place of delivery, family size and mother's age group showed significant impact only in rural areas while considering crude and adjusted odds.

**Table 4:** Crude odds ratio for effects of sociodemographic and maternal factors on low birth weight in Rural India using binary logistic regression

		Regions												
Explanatory Factors	India		Central		East		North		Northeast		South		West	
	COR (95% CI)	*p-*value	COR (95% CI)	*p-*value	COR (95% CI)	*p-*value	COR (95% CI)	*p-*value	COR (95% CI)	*p-*value	COR (95% CI)	*p-*value	COR (95% CI)	*p-*value
Religion (Hindu ®)														
Muslim	0.91 (0.88, 0.95)	< 0.001	1.08 (1.01, 1.16)	0.033	0.89 (0.83, 0.95)	0.001	0.82 (0.73, 0.93)	0.002	0.96 (0.83, 1.11)	0.583	0.89 (0.79, 1.02)	0.086	0.82 (0.70, 0.96)	0.016
Christian	0.84 (0.76, 0.92)	< 0.001	0.67 (0.34, 1.32)	0.243	1.14 (0.91, 1.44)	0.252	2.07 (1.21, 3.56)	0.008	0.58 (0.46, 0.73)	< 0.001	1.13 (0.98, 1.30)	0.083	0.69 (0.32, 1.49)	0.345
Other	1.15 (1.06, 1.25)	0.001	0.55 (0.34, 0.90)	0.016	1.03 (0.82, 1.29)	0.805	1.26 (1.13, 1.41)	< 0.001	0.66 (0.41, 1.06)	0.086	1.52 (0.73, 3.13)	0.261	1.21 (1.00, 1.47)	0.045
Social category (General ®)														
Schedule tribe	1.09 (1.04, 1.15)	< 0.001	0.98 (0.89, 1.08)	0.675	1.11 (1.01, 1.22)	0.036	1.32 (1.16, 1.50)	< 0.001	0.55 (0.43, 0.72)	< 0.001	0.94 (0.79, 1.12)	0.513	1.42 (1.27, 1.59)	< 0.001
Schedule caste	1.13 (1.08, 1.18)	< 0.001	1.05 (0.97, 1.14)	0.212	1.17 (1.08, 1.27)	< 0.001	1.25 (1.13, 1.39)	< 0.001	1.04 (0.78, 1.39)	0.779	1.07 (0.94, 1.22)	0.314	1.18 (1.04, 1.34)	0.012
OBC	1.00 (0.96, 1.04)	0.963	0.97 (0.91, 1.05)	0.474	1.02 (0.94, 1.10)	0.648	1.05 (0.95, 1.16)	0.382	1.02 (0.80, 1.32)	0.851	0.91 (0.80, 1.03)	0.130	1.05 (0.94, 1.17)	0.366
Birth order (1st ®)														
2nd	0.89 (0.87, 0.92)	< 0.001	0.91 (0.86, 0.96)	0.001	0.87 (0.82, 0.93)	< 0.001	0.96 (0.89, 1.05)	0.393	0.95 (0.81, 1.11)	0.518	0.82 (0.76, 0.89)	< 0.001	0.91 (0.83, 0.99)	0.033
3rd	0.89 (0.86, 0.93)	< 0.001	0.94 (0.88, 1.01)	0.088	0.73 (0.68, 0.79)	< 0.001	0.99 (0.89, 1.11)	0.920	1.01 (0.82, 1.26)	0.896	0.85 (0.76, 0.95)	0.004	0.99 (0.88, 1.11)	0.820
≥ 4th	0.92 (0.88, 0.96)	< 0.001	1.00 (0.93, 1.07)	0.970	0.76 (0.70, 0.83)	< 0.001	1.09 (0.96, 1.23)	0.180	0.90 (0.72, 1.13)	0.374	0.76 (0.62, 0.93)	0.007	0.84 (0.72, 0.98)	0.022
Place of delivery (Institution ®)														
Home	1.23 (1.17, 1.29)	< 0.001	1.27 (1.17, 1.37)	< 0.001	1.14 (1.05, 1.24)	0.003	1.60 (1.35, 1.90)	< 0.001	1.01 (0.83, 1.23)	0.919	1.70 (1.37, 2.12)	< 0.001	1.09 (0.92, 1.29)	0.301
Family size (≤ 4 ®)														
> 4	0.92 (0.89, 0.95)	< 0.001	0.84 (0.80, 0.89)	< 0.001	0.87 (0.82, 0.91)	< 0.001	0.93 (0.85, 1.02)	0.114	0.91 (0.79, 1.05)	0.187	0.92 (0.85, 0.99)	0.020	1.06 (0.96, 1.16)	0.279
Wealth Index (Rich ®)														
Poor	1.32 (1.28, 1.37)	< 0.001	1.33 (1.25, 1.42)	< 0.001	1.62 (1.46, 1.80)	< 0.001	1.24 (1.15, 1.35)	< 0.001	1.59 (1.17, 2.16)	0.003	1.46 (1.34, 1.59)	< 0.001	1.44 (1.32, 1.58)	< 0.001
Middle	1.09 (1.04, 1.13)	< 0.001	1.06 (0.98, 1.15)	0.147	1.18 (1.05, 1.34)	0.008	1.03 (0.94, 1.14)	0.488	1.11 (0.77, 1.59)	0.573	1.22 (1.12, 1.33)	< 0.001	1.19 (1.08, 1.32)	0.001
Mother's age group (35–49 ®)														
15–24 yrs	1.19 (1.13, 1.26)	< 0.001	1.09 (1.00, 1.19)	0.056	1.32 (1.18, 1.47)	< 0.001	1.10 (0.95, 1.27)	0.209	1.40 (1.10, 1.77)	0.007	1.11 (0.96, 1.29)	0.166	1.57 (1.32, 1.89)	< 0.001
25–34 yrs	1.00 (0.95, 1.06)	0.954	0.93 (0.85, 1.01)	0.095	1.02 (0.91, 1.14)	0.788	0.94 (0.81, 1.08)	0.351	1.25 (0.99, 1.58)	0.061	0.98 (0.85, 1.14)	0.798	1.26 (1.06, 1.51)	0.009
Intended pregnancy (Wanted then ®)														
Wanted later	1.17 (1.10, 1.24)	< 0.001	1.16 (1.04, 1.30)	0.008	1.26 (1.15, 1.38)	< 0.001	1.15 (0.98, 1.35)	0.079	1.07 (0.75, 1.52)	0.726	1.06 (0.87, 1.30)	0.561	1.06 (0.86, 1.31)	0.581
Wanted no more	1.11 (1.04, 1.18)	0.001	1.07 (0.96, 1.20)	0.232	1.05 (0.94, 1.18)	0.346	1.28 (1.07, 1.53)	0.006	0.92 (0.64, 1.32)	0.642	1.16 (0.92, 1.47)	0.195	1.31 (1.07, 1.61)	0.010
Mother's education (Higher ®)														
No education	1.48 (1.41, 1.56)	< 0.001	1.55 (1.42, 1.69)	< 0.001	1.47 (1.31, 1.65)	< 0.001	1.46 (1.29, 1.66)	< 0.001	1.69 (1.17, 2.46)	0.005	1.42 (1.24, 1.62)	< 0.001	1.48 (1.27, 1.74)	< 0.001
Primary	1.48 (1.40, 1.56)	< 0.001	1.59 (1.44, 1.74)	< 0.001	1.45 (1.28, 1.65)	< 0.001	1.43 (1.25, 1.65)	< 0.001	1.45 (1.00, 2.11)	0.048	1.41 (1.22, 1.64)	< 0.001	1.50 (1.27, 1.77)	< 0.001
Secondary	1.34 (1.28, 1.40)	< 0.001	1.42 (1.31, 1.54)	< 0.001	1.39 (1.24, 1.56)	< 0.001	1.30 (1.15, 1.46)	< 0.001	1.34 (0.95, 1.90)	0.092	1.29 (1.17, 1.41)	< 0.001	1.33 (1.16, 1.52)	< 0.001
Received full ANC (Yes ®)														
No	1.16 (1.12, 1.20)	< 0.001	1.23 (1.14, 1.32)	< 0.001	1.00 (0.94, 1.07)	0.974	1.33 (1.21, 1.47)	< 0.001	1.22 (1.02, 1.46)	0.029	1.08 (0.99, 1.17)	0.074	1.14 (1.04, 1.25)	0.005
Timing of first ANC visit (1st trimester ®)														
2nd trimester	1.06 (1.02, 1.10)	0.005	1.03 (0.97, 1.10)	0.376	0.98 (0.91, 1.05)	0.547	1.21 (1.08, 1.35)	0.001	1.03 (0.86, 1.22)	0.776	1.08 (0.96, 1.22)	0.218	1.05 (0.93, 1.19)	0.408
3rd trimester	1.09 (1.01, 1.16)	0.022	1.08 (0.95, 1.22)	0.238	0.99 (0.85, 1.15)	0.888	1.46 (1.16, 1.83)	0.001	1.12 (0.78, 1.59)	0.543	1.07 (0.91, 1.26)	0.411	1.08 (0.89, 1.31)	0.457
Mother's BMI (Normal ®)														
Underweight	1.29 (1.25, 1.34)	< 0.001	1.24 (1.17, 1.31)	< 0.001	1.38 (1.30, 1.46)	< 0.001	1.31 (1.19, 1.43)	< 0.001	1.46 (1.23, 1.74)	< 0.001	1.26 (1.15, 1.38)	< 0.001	1.21 (1.11, 1.32)	< 0.001
Overweight & Obese	0.86 (0.83, 0.90)	< 0.001	0.91 (0.84, 0.97)	0.008	0.82 (0.75, 0.89)	< 0.001	0.88 (0.79, 0.97)	0.012	0.66 (0.51, 0.85)	0.001	0.89 (0.82, 0.97)	0.009	0.89 (0.79, 1.00)	0.059
Mother's Anemia (Non anemic ®)														
Anemic	1.06 (1.03, 1.09)	< 0.001	1.09 (1.04, 1.15)	< 0.001	1.07 (1.01, 1.13)	0.017	1.07 (0.99, 1.15)	0.081	1.17 (1.01, 1.36)	0.033	1.00 (0.93, 1.08)	0.942	1.05 (0.97, 1.14)	0.237

*® Reference category*

**Table 5:** Adjusted odds ratio for effects of sociodemographic and maternal factors on low birth weight in Rural India using binary logistic regression

				Regions												
Explanatory Factors	India			Central		East		North			Northeast		South		West	
	AOR (95% CI)	*p-*value		AOR (95% CI)	*p-*value	AOR (95% CI)	*p-*value	AOR (95% CI)	*p-*value		AOR (95% CI)	*p-*value	AOR (95% CI)	*p-*value	AOR (95% CI)	*p-*value
Religion (Hindu ®)																
Muslim	0.97 (0.92, 1.03)	0.335		1.11 (1.00, 1.23)	0.041	0.94 (0.84, 1.04)	0.215	0.87 (0.73, 1.03)	0.100		0.66 (0.42, 1.03)	0.065	1.03 (0.87, 1.21)	0.748	0.89 (0.70, 1.14)	0.358
Christian	0.87 (0.77, 0.98)	0.023		0.68 (0.30, 1.56)	0.366	1.06 (0.79, 1.42)	0.688	3.02 (1.56, 5.83)	0.001		0.85 (0.59, 1.23)	0.400	1.10 (0.92, 1.32)	0.276	0.50 (0.16, 1.57)	0.237
Other	1.26 (1.14, 1.40)	< 0.001		0.46 (0.24, 0.88)	0.018	0.93 (0.69, 1.25)	0.633	1.43 (1.23, 1.66)	< 0.001		0.62 (0.31, 1.24)	0.174	1.11 (0.41, 2.97)	0.843	1.68 (1.30, 2.16)	< 0.001
Social category (General ®)																
Schedule tribe	0.90 (0.85, 0.97)	0.003		0.85 (0.74, 0.96)	0.011	0.85 (0.74, 0.97)	0.014	1.02 (0.85, 1.22)	0.841		0.47 (0.32, 0.70)	< 0.001	0.82 (0.66, 1.02)	0.078	1.29 (1.11, 1.50)	0.001
Schedule caste	1.04 (0.98, 1.09)	0.213		0.95 (0.86, 1.05)	0.323	1.02 (0.91, 1.14)	0.734	1.08 (0.94, 1.23)	0.268		0.86 (0.59, 1.25)	0.433	1.00 (0.85, 1.17)	0.966	1.04 (0.87, 1.24)	0.651
OBC	0.95 (0.91, 1.00)	0.061		0.90 (0.82, 0.98)	0.017	0.96 (0.87, 1.06)	0.378	1.00 (0.88, 1.13)	0.995		0.83 (0.60, 1.16)	0.275	0.89 (0.76, 1.04)	0.136	1.07 (0.94, 1.22)	0.315
Birth order (1st ®)																
2nd	0.84 (0.81, 0.88)	< 0.001		0.84 (0.78, 0.91)	< 0.001	0.80 (0.74, 0.87)	< 0.001	0.99 (0.89, 1.11)	0.882		0.96 (0.75, 1.22)	0.725	0.77 (0.69, 0.85)	< 0.001	0.84 (0.75, 0.95)	0.004
3rd	0.82 (0.78, 0.87)	< 0.001		0.86 (0.77, 0.94)	0.002	0.61 (0.54, 0.69)	< 0.001	1.04 (0.89, 1.21)	0.663		1.17 (0.81, 1.70)	0.401	0.83 (0.72, 0.96)	0.011	0.92 (0.78, 1.08)	0.310
≥ 4th	0.81 (0.76, 0.87)	< 0.001		0.90 (0.81, 1.01)	0.075	0.63 (0.55, 0.73)	< 0.001	1.04 (0.87, 1.26)	0.652		1.09 (0.67, 1.75)	0.740	0.69 (0.54, 0.88)	0.003	0.69 (0.55, 0.87)	0.002
Place of delivery (Institution ®)																
Home	1.19 (1.12, 1.28)	< 0.001		1.32 (1.19, 1.46)	< 0.001	1.10 (0.97, 1.24)	0.140	1.44 (1.13, 1.84)	0.003		1.10 (0.77, 1.57)	0.617	1.53 (1.12, 2.10)	0.007	0.85 (0.66, 1.08)	0.175
Family size (≤ 4 ®)																
> 4	0.94 (0.91, 0.98)	0.003		0.89 (0.83, 0.96)	0.003	0.92 (0.85, 0.99)	0.024	0.88 (0.79, 0.99)	0.037		0.84 (0.67, 1.04)	0.107	0.93 (0.85, 1.02)	0.144	1.07 (0.94, 1.21)	0.295
Wealth Index (Rich ®)																
Poor	1.20 (1.14, 1.26)	< 0.001		1.15 (1.06, 1.26)	0.002	1.57 (1.36, 1.81)	< 0.001	1.11 (0.98, 1.26)	0.115		1.31 (0.84, 2.03)	0.236	1.34 (1.18, 1.51)	< 0.001	1.30 (1.14, 1.49)	< 0.001
Middle	1.04 (0.99, 1.10)	0.103		1.00 (0.90, 1.10)	0.959	1.14 (0.98, 1.33)	0.101	0.99 (0.87, 1.12)	0.814		1.02 (0.63, 1.65)	0.936	1.18 (1.06, 1.31)	0.002	1.14 (1.00, 1.31)	0.045
Mother's age group (35–49 ®)																
15–24 yrs	1.11 (1.03, 1.20)	0.005		1.16 (1.02, 1.33)	0.023	1.02 (0.87, 1.19)	0.833	1.08 (0.88, 1.32)	0.447		1.26 (0.84, 1.89)	0.262	1.00 (0.83, 1.21)	0.988	1.45 (1.15, 1.83)	0.002
25–34 yrs	1.00 (0.94, 1.07)	0.948		1.03 (0.92, 1.16)	0.590	0.94 (0.81, 1.08)	0.371	0.96 (0.81, 1.15)	0.687		1.09 (0.77, 1.55)	0.614	1.01 (0.85, 1.20)	0.913	1.13 (0.91, 1.40)	0.278
Intended pregnancy (Wanted then ®)																
Wanted later	1.15 (1.06, 1.25)	0.001		1.15 (0.99, 1.35)	0.074	1.27 (1.11, 1.44)	< 0.001	1.22 (0.98, 1.51)	0.081		1.27 (0.75, 2.15)	0.372	1.06 (0.81, 1.39)	0.681	0.86 (0.63, 1.16)	0.319
Wanted no more	1.12 (1.02, 1.22)	0.016		1.05 (0.90, 1.23)	0.534	1.14 (0.96, 1.34)	0.133	1.18 (0.92, 1.51)	0.197		0.75 (0.39, 1.44)	0.381	1.22 (0.91, 1.63)	0.180	1.49 (1.12, 1.99)	0.007
Mother's education (Higher ®)																
No education	1.42 (1.33, 1.53)	< 0.001		1.43 (1.26, 1.61)	< 0.001	1.56 (1.32, 1.84)	< 0.001	1.24 (1.03, 1.49)	0.024		1.47 (0.84, 2.59)	0.181	1.31 (1.09, 1.58)	0.004	1.26 (1.00, 1.59)	0.046
Primary	1.46 (1.36, 1.57)	< 0.001		1.48 (1.31, 1.68)	< 0.001	1.44 (1.22, 1.71)	< 0.001	1.37 (1.14, 1.66)	0.001		1.28 (0.73, 2.23)	0.390	1.39 (1.14, 1.68)	0.001	1.43 (1.14, 1.80)	0.002
Secondary	1.29 (1.22, 1.37)	< 0.001		1.32 (1.19, 1.46)	< 0.001	1.33 (1.15, 1.54)	< 0.001	1.18 (1.02, 1.37)	0.028		1.11 (0.68, 1.81)	0.680	1.25 (1.11, 1.41)	< 0.001	1.30 (1.09, 1.54)	0.003
Received full ANC (Yes ®)																
No	1.09 (1.05, 1.13)	< 0.001		1.20 (1.11, 1.30)	< 0.001	0.96 (0.89, 1.03)	0.261	1.27 (1.14, 1.41)	< 0.001		1.10 (0.88, 1.38)	0.403	1.02 (0.93, 1.11)	0.675	1.08 (0.98, 1.19)	0.143
Timing of first ANC visit (1st trimester ®)																
2nd trimester	1.03 (0.99, 1.07)	0.205		0.99 (0.93, 1.06)	0.784	1.02 (0.94, 1.10)	0.672	1.13 (1.00, 1.27)	0.053		1.07 (0.85, 1.35)	0.551	1.04 (0.91, 1.18)	0.550	1.07 (0.94, 1.22)	0.282
3rd trimester	1.06 (0.99, 1.14)	0.119		1.04 (0.92, 1.19)	0.523	0.97 (0.82, 1.14)	0.691	1.38 (1.08, 1.75)	0.009		1.10 (0.71, 1.70)	0.661	1.08 (0.92, 1.28)	0.356	1.01 (0.82, 1.25)	0.894
Mother's BMI (Normal ®)																
Underweight	1.24 (1.19, 1.29)	< 0.001		1.17 (1.09, 1.26)	< 0.001	1.29 (1.19, 1.39)	< 0.001	1.31 (1.16, 1.47)	< 0.001		1.32 (1.03, 1.69)	0.031	1.24 (1.11, 1.38)	< 0.001	1.18 (1.06, 1.32)	0.003
Overweight & Obese	0.91 (0.87, 0.96)	< 0.001		0.98 (0.89, 1.07)	0.591	0.85 (0.76, 0.96)	0.006	0.92 (0.81, 1.05)	0.235		0.79 (0.57, 1.10)	0.168	0.89 (0.80, 0.99)	0.033	0.89 (0.77, 1.04)	0.155
Mother's Anemia (Non anemic ®)																
Anemic	1.01 (0.98, 1.05)	0.525		1.08 (1.02, 1.15)	0.011	1.04 (0.97, 1.12)	0.276	1.00 (0.91, 1.09)	0.926		1.11 (0.89, 1.38)	0.365	0.95 (0.88, 1.04)	0.280	0.91 (0.83, 1.01)	0.073

*® Reference category*

In comparison to Hindu children, Muslims and Christians were more likely to have LBW in the rural central and north regions based on COR and AOR. LBW was more common among the other religious groups in the northern and western regions and also in the overall country (COR = 1.15, CI: 1.06–1.25, *p* = 0.001; AOR = 1.26, CI: 1.14–1.40, *p* < 0.001). According to AOR, the prevalence of LBW among the STs was less common in rural India, including central, east, and northeast regions but more prevalent in the west compared to the general caste category. The probability of LBW remained lower among children following the birth of the first child in rural India, including central, eastern, southern, and western regions based on both COR and AOR. The child who born at home were more likely to have LBW in rural India (COR = 1.23, CI: 1.17–1.29, *p* < 0.001; AOR = 1.19, CI: 1.12–1.28, *p* < 0.001) compare to institutional birth, including central, north, and south regions for both COR and AOR. Family size was a significant factor for LBW; the probability of LBW was lower in households with more than four people in rural India (COR = 0.92, CI: 0.89–0.95, *p* < 0.01; AOR = 0.94, CI: 0.91–0.98, *p* < 0.01), including central, and eastern regions for both COR and AOR. Mothers from poor families were more likely to have LBW children than rich families in rural India, including central, east, south and west for both COR and AOR.

The LBW children were more likely found among the mothers aged 15–24 years compared to aged 35–49 years in rural India (COR = 1.19, CI: 1.13–1.26, *p* < 0.01; AOR = 1.11, CI: 1.03–1.20, *p* < 0.01), including western regions based on COR and AOR. Unintended pregnancies significantly increased the chances of LBW in east and west India for both COR and AOR. Mothers with lower levels of education were more likely to have LBW children in and across India compared to higher-educated mothers, except for the northeastern region in AOR. The risk of LBW was higher among mothers who did not receive full ANC in overall rural India including central and northern regions based on COR and AOR. In northern region, the risk of LBW was significantly increased when mothers visit for the first ANC after 1st trimester in rural north India both for COR and AOR. LBW more likely found among the underweight mothers in and across rural regions of India both for COR and AOR. Based on COR and AOR, maternal anaemia increase the chances of LBW in rural central India (COR = 1.09, CI: 1.04–1.15, *p* < 0.01; AOR = 1.08, CI: 1.02–1.15, *p* = 0.011).

## Area under the ROC curve: an impact of explanatory factors on LBW

**Fig. 3:**
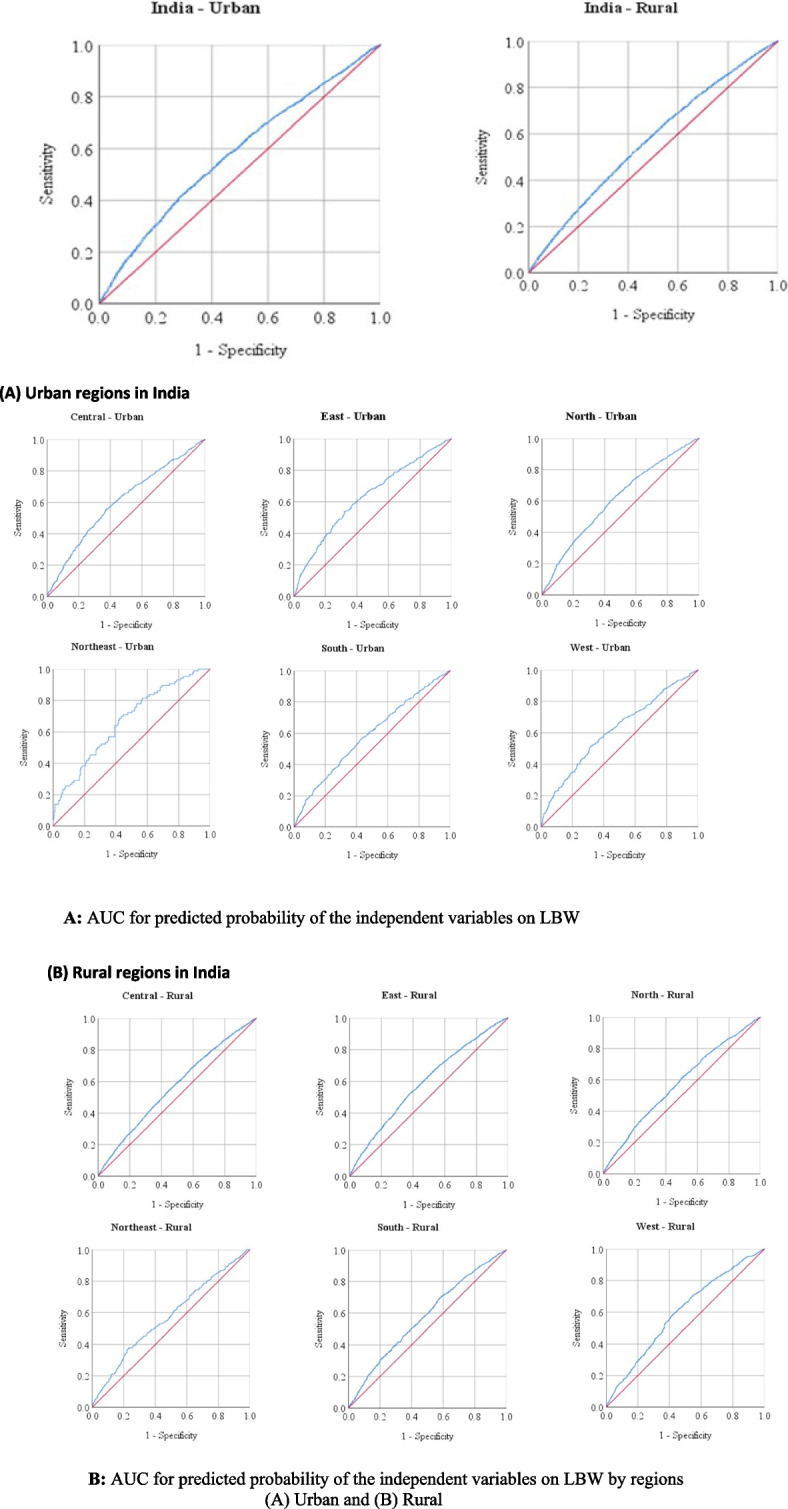
**A** AUC for predicted probability of the independent variables on LBW. **B** AUC for predicted probability of the independent variables on LBW by regions. **A** Urban and (**B**) Rural

The overall effect of the predicted probability of independent variables on LBW, which is vary by region shown in Table 6 and the ROC curve below (Fig. 3A, B). Predicted probability levels showed the cumulative impact of all independent variables on the dependent variable, known as LBW. The independent variables had a significant impact (AUC, *p* < 0.001) on LBW because the AUC of predicted probability for the independent variables in each curve was larger than 50%. In the urban area of northeast India, with an AUC value of 67%, and the rural area of west India with an AUC value of 60%, the predicted probability of independent variables had the maximum impact. However, the AUC shows that 58% and 57% cumulative effects of independent variables on LBW can be distinguished in urban and rural India, respectively. Youden’s *J* indicates there was a minor but discernible impact of the explanatory variables on LBW.

**Table 6:** Area under curve (AUC) based on the impact of predicted probability on LBW

Country/ Regions	Urban			Rural		
	AUC (95% CI)	*p-*value	*J*-value	AUC (95% CI)	*p-*value	*J*-value
India	0.58 (0.57, 0.59)	< 0.001	0.13	0.57 (0.56, 0.57)	< 0.001	0.10
Central	0.60 (0.59, 0.62)	< 0.001	0.18	0.57 (0.56, 0.58)	< 0.001	0.10
East	0.63 (0.61, 0.65)	< 0.001	0.22	0.59 (0.59, 0.60)	< 0.001	0.14
North	0.61 (0.59, 0.62)	< 0.001	0.17	0.57 (0.56, 0.59)	< 0.001	0.11
Northeast	0.67 (0.61, 0.73)	< 0.001	0.26	0.58 (0.54, 0.61)	< 0.001	0.15
South	0.59 (0.57, 0.60)	< 0.001	0.14	0.58 (0.57, 0.59)	< 0.001	0.12
West	0.62 (0.60, 0.63)	< 0.001	0.20	0.60 (0.58, 0.61)	< 0.001	0.16

Under the nonparametric assumption

Null hypothesis: true area = 0.5

## Discussion

Low birth weight remains a public health challenge in LMICs, including India; thus its burden and determinants in different geographic regions need to be known to the stakeholders. The study indicated that although the prevalence of LBW has decreased from the NFHS-3 (2005–06) level of 21.5% to 18.24% in the NFHS-5, it has essentially remained stable from NFHS-4 (18.2%) to NFHS-5 [12, 20, 21]. The prevalence was comparatively higher in rural areas (18.58%) than its urban counterpart (17.36%) in NFHS-5, which was equivalent to NFHS-4 (Urban 17.6% and Rural 18.5%). Based on NFHS-5, the prevalence remains critical, especially for rural western (20.63%), central (20.16%) regions, and urban northern (19.20%), central (18.83%) regions.

Sociodemographic and maternal factors were found to be significantly associated with the prevalence of LBW regardless of rural or urban area. In both rural and urban areas, the social category was found to be a significant predictor for LBW, particularly for the Schedule Caste group. The other most common factors for urban and rural areas were pregnancies delivered at home and households with poor wealth index, similar trend has been found from the studies from sub-Saharan Africa, Ethiopia, Sri Lanka, and Bangladesh [34–37]. Other religious groups, such as Sikhs, Buddhists, and Jains, others were shown to have a substantial impact on LBW in rural regions under the social category factor; in contrast, 4th or higher birth orders were found to have a significant impact on LBW in urban areas. In contrast, in urban area 4th or higher birth order was found to have crucial impact on LBW.

Furthermore, it seems that in both urban and rural areas, maternal education and ANC become a common factor influencing LBW in the case of maternal variables. The study indicated that lower maternal education had a substantial impact on LBW in both rural and urban areas. Similar findings have shown that lower mother education levels and LBW of children coexist in a number of LMICs, including Ethiopia, Bangladesh, Kenya, Zambia, Pakistan, and Guatemala [34, 38–40]. Children with LBW were more prone to be born from unexpected pregnancies that were wanted later and from babies born to young mothers. Studies conducted in other LMICs, including Kenya, Brazil, and Turkey, have shown the effectiveness of lower mother ages in LBW [39–42]. Other influential factors that increased the risk of LBW in children were maternity care services. Mothers who did not receive full ANC and visited ANC after 1st trimester showed an increased prevalence of LBW, indicating that the ANC had a significant impact on LBW. Studies conducted in Brazil and Bangladesh also shown that the LBW and the lack of ANC coexisted [41, 43].

Regionally, Christians were four times more prone to have LBW in urban central and western India, and other religious groups were almost five times more likely in the Southern region. In contrast, Christians from the South and Muslims from the Central region had higher rates of LBW in rural areas. Therefore, this disparity could be influenced by religious practises, which varies across regions in India. The backward section of contemporary society, including SCs in the eastern, OBCs in the southern urban areas, and STs in the western rural areas, had a disproportionately high risk of having LBW children. Birth order was a significant risk factor that enhanced the incidence of LBW in the northern region. The chance of LBW was more significant for mothers in the western region who delivered at home. Some studies suggested that inappropriate health-seeking behaviour and a lack of access to improved healthcare facilities may indirectly impact a child's birth weight [44].

The current study shows that mothers from low- and middle-income groups in rural and urban areas were more likely to deliver children with LBW. Additionally, the LBW of children was found to be associated with mothers’ anaemia levels and nutritional status (BMI). Underweight mothers were more vulnerable to have LBW in all regions, particularly in urban northeastern regions. Similarly, in urban northeast and rural central region anaemic level of mothers have higher impact on LBW. The availability of public health professionals, infrastructure, modern health care facilities, and public awareness varied by regions in India [45, 46]. This seems to be a contributing factor to the regional difference in LBW. However, some factors that coexisted consistently with LBW from NFHS-3 to NFHS-5 and persisted across geographies, such as maternal education, nutritional status, antenatal care, and a poor wealth index, need to be addressed in order to reduce the prevalence of LBW in India [19, 47]. The present study revealed a consistent trend to that observed in NFHS-4, where poor household wealth index, low maternal education in the central and eastern region were more prevalent and had a substantial impact on LBW [22].

### Strengths and limitations of the study

The study has precisely presented the region-wise picture of low birth weight children with its determinants based on the most recent DHS Survey of India, conducted between 2019 and 2021. This provides an opportunity to develop region-specific policies or revise the existing policies to address the age-old problem of LBW in India. The study highlighted regional division because each region has differences based on geographic location, language, culture, climate, ethnicity, and historical context that separate one region from another. The values of AUC and Youden J index indicates that the predicted probability of the selected independent variables on LBW was substantial but low. So, there may be some potential confounders apart from the studied variables which may have effect on LBW. In our next study, we will aim to identify those factors, which could include ecological patterns, epidemiological trends, social norms, values, traditions, and practises. Additionally, if a similar kind of study can be conducted on each state, then state-specific policies can be developed in the country. However, more studies and research needs to be undertaken to understand the holistic picture of various issues particularly associated with low birth weight in India. We did not check the variation of our outcome variable among different regions in India, if the there was a variation, the multilevel logistic regression model was more appropriate. Furthermore, in this study efforts have been made to capture the various factor’s associations and comparisons in different layers of regional divisions. Hence, it paved the way with a substantial contribution towards exploring and understanding varied factors contributing towards the stagnant burden of low birth weight in India.

## Conclusion

There has been a gradual decline in the burden of LBW in India over a period of time, but there has been hardly any significant decline in its occurrence compared to NFHS-4 data. Thus targeted specific strategies need to be undertaken as per region and residential areas. Approaches should have been region and residential area specific. More emphasize needs to be given on addressing maternal health including nutritional status, improving maternal education and uplifting economic status that was found persistent form NFHS-3 to NFHS-5. Then only India should be able to reduce LBW as desired, which has also been emphasised by National Health Policy. The present study will promote the nation in achieving five of the United Nations’ seventeen Sustainable Development Goals: no poverty, zero hunger, good health and well-being, quality education, and reduced inequalities. A trend analysis is required as it appears that the LBW issue in the country has almost stagnated over the last 5 years.

## Data Availability

The NFHS-5 datasets are available at https://dhsprogram.com/Data/.

## Abbreviations

ANC: Antenatal Care
AOR: Adjusted Odd Ratio
AUC: Area Under Curve
BMI: Body mass index
CI: Confidence Interval
COR: Crude Odd Ratio
DHS: Demographic and Health Surveys
IFA: Iron Folic Acid
LBW: Low Birth Weight
LMICs: Low- and Middle-Income Countries
NFHS-5: National Family Health Survey, India 2019–21
NLBW: Non-Low Birth Weight
OBC: Other Backward Classes
ROC: Receiver Operator Characteristic
ROC: Receiver Operator Characteristic
SC: Scheduled caste
SDGs: Sustainable Development Goals
SPSS: Statistical Package for the Social Sciences
ST: Scheduled tribe
TT: Tetanus Toxoid
UNICEF: United Nations Children'’ Fund; Maternal, Newborn, Child and Adolescent Health
VIF: Variance inflation factor
WHO: World health organization
